# RELATING THE ORGANIZATIONAL CULTURE ASSESSMENT INSTRUMENT TO ADHERENCE IN A PRAGMATIC TRIAL OF A MUSIC INTERVENTION

**DOI:** 10.1093/geroni/igad104.2135

**Published:** 2023-12-21

**Authors:** Enya Zhu, Ellen McCreedy, Laura Dionne, Vincent Mor

**Affiliations:** Brown University School of Public Health, Providence, Rhode Island, United States; Brown University School of Public Health, Providence, Rhode Island, United States; Brown University School of Public Health, Providence, Rhode Island, United States; Brown University School of Public Health, Providence, Rhode Island, United States

## Abstract

Embedded pragmatic trials encourage the translation of evidence-based interventions to “real-world” settings. Most pragmatic trials of behavioral interventions for people with dementia suffer from low adherence. Understanding how organizational values and structure may increase adherence is important. We report findings from an embedded, pragmatic trial (ePCT) of a personalized music intervention for managing behaviors in residents with dementia, conducted in 54 nursing homes (NHs) from four corporations between June 2019 and February 2020. Before the trial began, the administrator and a nursing staff member from each NH completed the Organizational Culture Assessment Instrument (OCAI). Using the OCAI, respondents rated their organizational culture by allocating a total of 100 points across four competing domains: Clan, Adhocracy, Hierarchy, and Market. Results were aggregated to understand how differences in culture impacted corporate-level adoption of the intervention. All four corporations allocated the majority of their points to Clan culture, which is focused on collaboration and staff engagement. However, corporations differed in their scoring of the secondary culture type. The two corporations that rated Hierarchical culture, which prioritizes consistency and efficiency, highly were more likely to adhere to the intervention protocols. The corporation with Market highly rated had the lowest adherence to the protocols. After controlling for other corporate characteristics, including for-profit status, size, and overall quality, hierarchical culture was associated with greater numbers of exposed residents and a higher dose of the music, compared to other culture types. Understanding the role of organizational culture on pragmatic implementation is an understudied area for research.

